# miRNA-regulated dynamics in circadian oscillator models

**DOI:** 10.1186/1752-0509-3-45

**Published:** 2009-05-05

**Authors:** Amitabha Nandi, Candida Vaz, Alok Bhattacharya, Ramakrishna Ramaswamy

**Affiliations:** 1 Center for Computational Biology and Bioinformatics, School of Information Technology, Jawaharlal Nehru University, New Delhi 110067, India; 2 School of Life Sciences, Jawaharlal Nehru University, New Delhi 110067, India; 3 School of Physical Sciences, Jawaharlal Nehru University, New Delhi 110067, India

## Abstract

**Background:**

We have studied the dynamics of miRNA regulation in two models of circadian oscillators. miRNAs are a class of small RNA molecules (18–24 nucleotides) that are known to regulate gene expression at the post-transcriptional level by reducing the amount of proteins produced by translation. This is done either by blocking translation or by degradation of mRNAs, the latter being mainly due to the initiation of a set of processes induced by formation of the miRNA:mRNA complex. Although miRNAs are known to regulate a large number of fundamental biological processes such as growth and development, their role in the dynamics of regulation is not completely understood. In exceptional cases, in particular, they can also up-regulate gene expression.

**Results:**

We have studied simple biological systems wherein oscillations originate from negative auto regulation of gene expression. The regulation of gene expression by miRNAs is introduced into these models and the dynamics is studied via standard stochastic simulation techniques. We find that in addition to a reduction in the amplitude of the oscillation, inclusion of miRNAs in the models has the effect of altering the *frequency *of oscillation and thereby regulating the dynamics of protein production.

**Conclusion:**

miRNAs can have a profound effect on the dynamics of regulatory modules, both by control of amplitude, namely by affecting the level of gene expression, as well as by control or alteration of frequency, namely by interference with the temporal sequence of gene production or delivery. We believe that our results are valid for a variety of regulatory systems, beyond the exemplars discussed here.

## Background

Fresh insight into gene expression regulation has been brought in recent years from the discovery that microRNAs (miRNAs), a class of non-coding small RNAs of length about 22 nucleotides, can play a crucial role in the process. Although the precise role of miRNAs has not been fully elucidated, it is known that starting from a large transcript these are generated by a series of nuclease-mediated processing events [1,2], or by the processing of introns [3]. miRNAs are known to act as post-transcriptional gene suppressors: they act by base-pairing with their target mRNAs and inducing either translational repression or mRNA degradation through a RNA-induced silencing complex (RISC) [1,2]. It has also been reported that in exceptional cases miRNA can *up-regulate *gene expression [4] but the manner in which this happens is even less understood.

The first miRNAs to be identified were *let7 *and *lin4 *in *Caenorhabditis elegans *[5-7]. Advance in the area of small RNA research has been rapid, and by now several hundred miRNAs are known in eukaryotic organisms (including in a single-celled eukaryote [8]). These are found to participate in a variety of fundamental processes such as growth and differentiation [9], and their biogenesis, functionality and target gene regulation has been explored in detail. Dysregulation of miRNA biogenesis has been found to be widespread in a number of diseases, notable among which are a variety of cancers [10,11].

What is the dynamics of miRNA regulation at the microscopic level? This is the main focus of the present paper, wherein we study the temporal effects of miRNA regulation on genetic oscillators. A number of sub-cellular phenomena are known to display temporal oscillations, and their regulation is crucial, both in expression level (or amplitude) as well as in frequency or in relative phase. In particular, we focus on circadian oscillators: miRNAs are known to be implicated in processes that control cellular clocks [12-14] and there are well – developed mathematical models for such processes. The expression of many miRNA genes is also experimentally known to follow circadian cycling, making this a suitable system for modeling studies.

In order to understand quantitative aspect of miRNA-mediated regulation, we examine the dynamics of two different model circadian oscillators, and incorporate additional reaction channels for the interactions of miRNA with mRNAs. The two models differ in the manner in which oscillations are generated: one essentially involves predator – prey type dynamics, suitably modified in order to describe the interaction of activator and repressor genes, while the other is based on an auto – regulated negative feedback loop. We carry out a detailed study of the stochastic dynamics of these models and show that miRNA can affect both the amplitude and the frequency of oscillations. By varying the intrinsic parameters it is possible to induce a wide variation of both the gene expression level as well as its dynamics, so that complex temporal patterns can be achieved through a relatively small number of controls. In addition to the stochastic simulation results, we have also examined the corresponding deterministic models and find that our major conclusions are validated there as well (see Additional Files 1, 2, 3, 4, 5, 6 and 7 for details). We therefore believe that the primary features in our simulations apply more generally, and thus the effect of miRNA regulation in a range of sub-cellular processes is likely to be through the modification of both the level of gene expression as well as its variation in time.

## Results

The main objective of the present work is the study of miRNA post-transcriptional regulation dynamics. A number of plausible mechanisms for the action of miRNA at the microscopic level are applied to model genetic networks that have been recently studied in considerable detail. Simulations of these processes show that the dynamics of the coupled set of reactions can be significantly altered through miRNA-mediated control. While these models are quite general, our results are in consonance with recent experimental observations on the role of miRNAs in regulating circadian clocks and may therefore capture the main features of such regulation.

## Circadian Oscillator models

Environmental periodicity creates the need for organisms to develop a sense of internal time, and thus biological clocks have evolved a range of internal time-keeping mechanisms that generate circadian, ultradian or infradian rhythms. These oscillators are robust to temperature fluctuations that affect the rates of chemical reactions, and additional internal noise that derives from the stochastic nature of chemical reactions.

Elucidation of the mechanism of circadian clocks has been greatly facilitated by the identification of mutants and their cognate genes in Cyanobacteria, Drosophila, Neurospora and a number of other organisms [15]. Molecular and genetic studies indicate that a circadian period arises from a system of interconnected feedback loops that control the transcription of a small number of "clock" genes [16]. A small molecule like c-AMP can also participate in generation of cellular rhythms through signalling and transcriptional control [17]. While circadian rhythms in different species are outwardly similar, the genes that make up the clock mechanisms are quite different in animals, plants, fungi or in cyanobacteria. A crucial feature is the ability to maintain a constant period over a wide range of internal and external fluctuations. In a very general sense, negative feedback together with time – delay in the interaction is sufficient in principle to produce oscillations. However, different circadian clocks are composed of distinct organizational modules, and it remains a challenge to understand this complexity.

**Figure 1:**
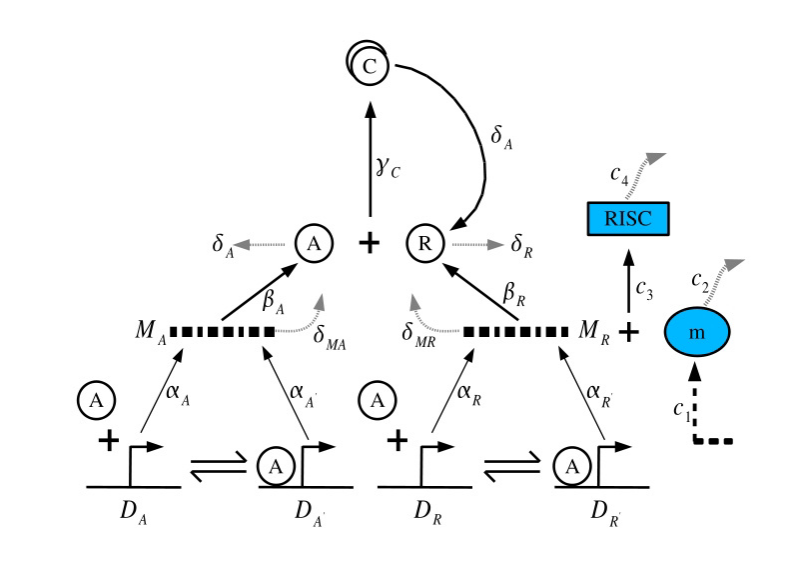
**Biochemical network of the extended circadian oscillator model (Model A) with miRNA regulation at the post-transcriptional level**. D_A_' and D_A _denote the number of activator genes with and without A bound to its promoter respectively, and D_R_', D_R _refer to the repressor gene driven by the common promoter A. M_A_, M_R _denote the mRNA corresponding to the activator A and the repressor R. C corresponds to the inactivated complexes formed by A and R with binding rate γ_c_. The constants α (α_A _and α_R_) and α' (α_A' _and α_R'_) denote the basal and activated rates of transcription, β (β_A _and β_R_) the rates of translation, δ (δ_A _and δ_R_) the rates of spontaneous degradation. The details of the reaction rate values are given in [18]. The miRNA m combines with M_R _to form the RISC *C*_*RISC*_. c_1 _is the rate of production of miRNA, c_2 _is its decay rate. c_3 _is the rate at which miRNA combines with R and c_4 _is rate at which RISC degrades. Throughout the volume is assumed to be unity. The cellular volume is assumed to be the unity so that concentrations and number of molecules are equivalent. It is assumed that the complex breaks into R because of the degradation of A and, therefore, the parameter δ_A _appears twice in the model.

Here we study regulation in two different circadian oscillator models. **Model A**, shown schematically in Fig. 1, has been quantitatively studied in detail [18]. This has all essential elements that have been found experimentally [19], and consists of an activator A and repressor R, that are transcribed and subsequently translated. The activator A increases its own transcription as well as that of the repressor by binding to the corresponding promoters: A thus acts as a positive element in transcription, whereas R is a negative element that sequesters the activator. The cycle completes itself upon degradation of the negative element and re-expression of the activator.

**Figure 2:**
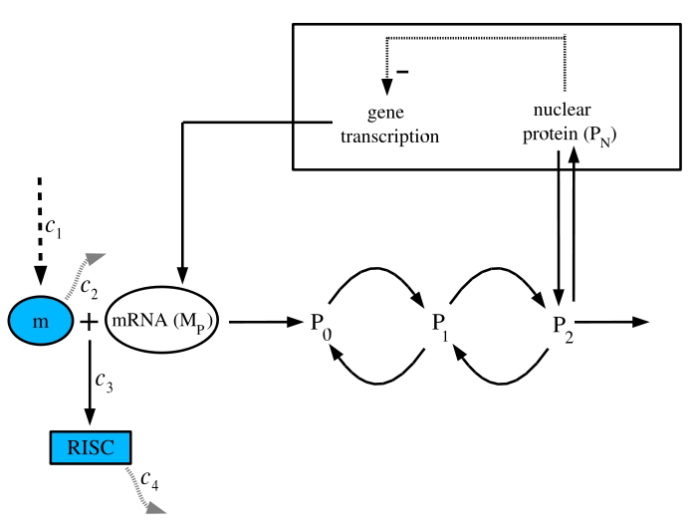
**Circadian Oscillator model (Model B) with miRNA regulation**. The model incorporates gene transcription, transport of mRNA (M_P_) into the cytosol where it is translated into the clock protein (P_0_) and degraded. The clock protein can be reversibly phosphorylated from the form P_0 _into the forms P_1 _and P_2_, successively. The latter form is degraded or transported into the nucleus (P_N_), where it exerts a negative feedback of cooperative nature on the expression of its gene. The model accounts for circadian oscillations of *Per *mRNA and corresponding protein in Drosophila. Details of the model are available in [20] and miRNA regulation is included in the model in a similar fashion as in Fig. 1

The second model (termed **Model B**) is based on the negative feedback exerted by a regulatory protein on the expression of its own gene. Such a negative regulatory mechanism underlies circadian oscillations of the PER protein in Drosophila and of the FRQ protein in Neurospora [20] and is shown schematically in Fig. 2. This incorporates gene transcription into mRNA, translation of mRNA into protein, reversible phosphorylation leading to degradation of the regulatory protein, transport of the latter into the nucleus, and repression of gene expression by the nuclear form of the protein.

## Incorporation of miRNA regulation in the models

Experiments have established that miRNAs act as fine-tuners of gene regulation: they directly affect levels of their target transcripts by accelerating their degradation rates [21] and thereby lower the expression levels. This is achieved through binding of miRNA by partial nucleotide sequence complementarity to the target mRNA sequences that helps the miRNA:mRNA duplex to be part of the multi-protein silencing or RISC (RNA induced silencing complex). Once mRNA reaches the RISC it is not available for translation; this causes a reduction in the expression of the encoded protein.

There are two possible outcomes of this process. Either the mRNAs get degraded, or the RISC dissociates, releasing mRNAs which can then add to the pool of mRNAs waiting to be translated. In addition to the elementary processes that are intrinsic to the oscillator models, the additional reaction channels that need to be incorporated when considering miRNA regulation therefore are

(1)

(2)

(3)

(4)

where **m **denotes the miRNA, **M **the mRNA associated with the gene expression and **C**_**RISC **_denotes the **RISC **complex. Eqs. (1) and (2) imply that the introduction or degradation of miRNA in the system is at a constant rate, while Eqs. (3) and (4) represents the formation and degradation of the RISC. The exact functional form of the miRNA-mediated degradation of its target mRNA is not known except that the presence of miRNA enhances the degradation. We make the plausible assumption here that the degradation rate of a target mRNA is proportional to the miRNA level, and thus in the present model Eqs. (1–4) represents the miRNA regulatory process at the post-transcriptional level.

An alternate channel that may be operative in some cases pertains to the miRNA mediated degradation process. If the RISC can release mRNA and miRNA, then Eq. (4) should be replaced by

(5)

The studies reported here are based on gene regulation through Eqs. (1–3) and either Eq. 4 or Eq. 5. These are termed ***Case 1 ***(mRNAs degraded in the RISC) and ***Case 2 ***(mRNAs released from the RISC) respectively. One way to incorporate the time scale separation between the formation of RISC and the release of m and M is to include time-delay **τ in **Eq. 5. Further, it is also plausible that the miRNA is introduced in the system as a function of the gene product,

(6)

For simplicity we have considered only linear dependence, and we also do not include time delay in our simulations in the present work.

Details of the elementary processes that constitute the two circadian oscillators, Models A & B have been discussed at length earlier. In Model A [18,19] we consider the repressor gene R to be under regulation (see Fig. 1) while in Model B, we take PER to be the regulated protein [20].

## Model A [18,19]

The temporal behavior of the Repressor **R **under miRNA control is studied under the two conditions described above. In general, incorporation of miRNA enhances the degradation of repressor mRNA, and therefore it can be anticipated that there will be corresponding effect on the dynamics.

### Case1: Degradation of mRNA in the RISC

**Figure 3:**
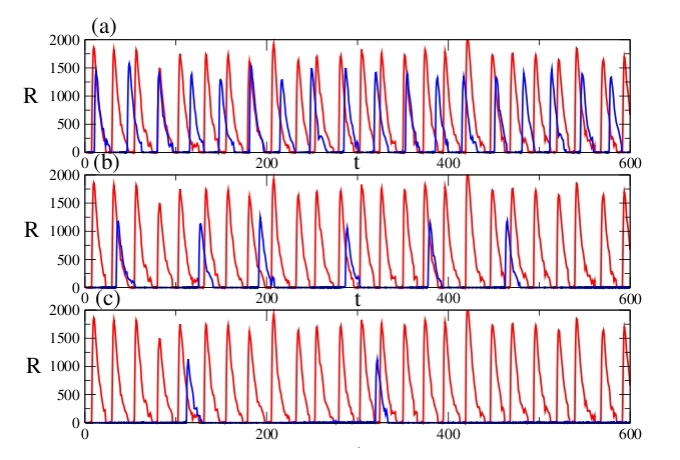
**Model A, Case 1: Temporal behavior of the repressor protein R with (blue) and without (red) miRNA regulation**. The degradation rate of the miRNA is fixed at c_2 _= 0.029, and the volume is taken to be unity in all cases. Regulation with (a) c_1 _= 20 h^-1^, c_3 _= 6 h^-1 ^and c_4 _= 0.6 h^-1^, (b) c_1 _= 40 h^-1^, c_3 _= 10 h^-1 ^and c_4 _= 1 h^-1^, and (c) c_1 _= 45 h^-1^, c_3 _= 20 h^-1 ^and c_4 _= 3 h^-1^.

In addition to translation blocking [22], miRNA – induced degradation of mRNAs can be a major mechanism for the reduction in gene expression. The results of simulation with different values of the specific rate constants are shown in Fig. 3. The red curves indicate the variation of protein **R **in absence of miRNA. On introduction of miRNA, the oscillatory behaviour changes significantly, with a reduction in both amplitude and frequency (shown in blue).

### Case2: RISC releases miRNA and mRNA

**Figure 4:**
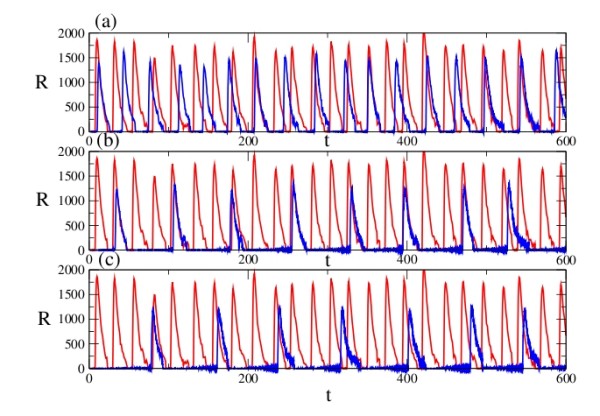
**Model A, Case 2: Temporal behaviour of the repressor protein R with (blue) and without (red) miRNA regulation**. The parameters are identical to those in Fig. 3, except that c_4 _here corresponds to the rate of dissociation of the RISC complex.

The results of simulations for this scenario are shown in Fig. 4. The decrease in frequency and amplitude was not as large as that observed in Case 1. Compared to Case 1, the temporal behavior shown in Case 2 appears to have a higher level of stability. There may be a correlation between this latter mechanism and environmental stress: it is known, for instance, that amino acid transporter CAT-1 mRNA is reversibly released from the RISC under such conditions [23].

### The effect of parameter variation

**Figure 5:**
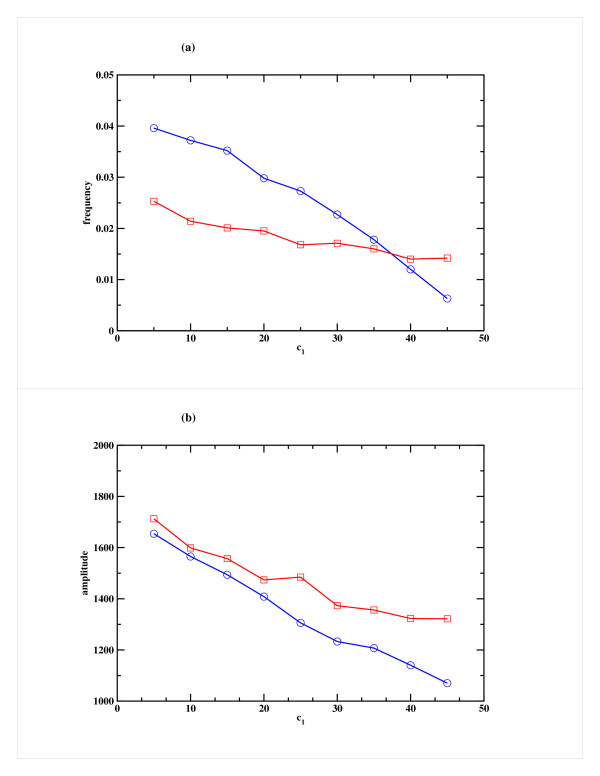
**Effect of variation of the miRNA production rate (c_1_) on the repressor R for Model A**. Variation of (a) the frequency and (b) the amplitude of oscillation in the Repressor as a function of c_1_, the rate of miRNA production. Cases 1 and 2 are shown in blue and red respectively. The other parameters, namely the rate of binding of miRNA to mRNA (c_3_) and RISC degradation rate (c_4_) are both kept fixed at 1 h^-1^.

In order to verify that the observed dynamical effects are robust to parameter variation, we have carried out some exploration of the parameter space. The extent to which the frequency is reduced is dependent on the rate of miRNA mediated down-regulation of repressor gene expression. When the rate of production of miRNA (c_1_) is increased, this reduction can be quite dramatic as shown in Fig. 5a for a typical case. Here c_1 _was increased keeping the other rate constants fixed. The corresponding decrease in amplitude is shown in Fig. 5b. For the Case 2 scenario, the decrease in frequency and amplitude is less pronounced.

**Figure 6:**
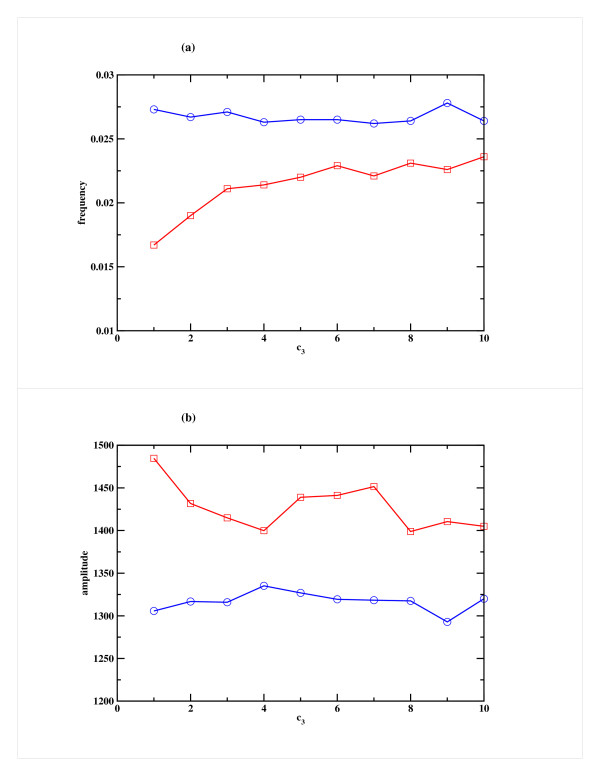
**Effect of variation of the rate of binding of miRNA to mRNA (c_3_) on the repressor R for Model A**. Variation of (a) the frequency and (b) the amplitude of oscillation in the Repressor as a function of c_3_, the rate of binding of the miRNA to mRNA. Cases 1 and 2 are shown in blue and red respectively. The other parameters, namely the miRNA production rate (c_1_) and RISC degradation rate (c_4_) are kept fixed at 25 h^-1 ^and 1 h^-1 ^respectively.

**Figure 7:**
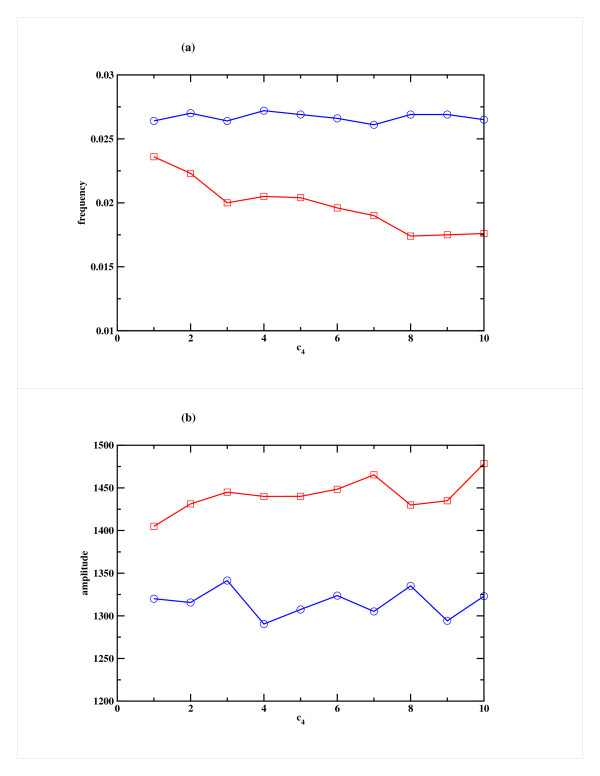
**Effect of variation of the RISC degradation rate (c_4_) on the repressor R for Model A**. Variation of (a) the frequency and (b) the amplitude of oscillation in the Repressor as a function of c_4_, the RISC degradation rate. Cases 1 and 2 are shown in blue and red respectively. The other parameters, namely the miRNA production rate (c_1_) and the rate of binding of miRNA to mRNA (c_3_) are kept fixed at 25 h^-1 ^and 10 h^-1 ^respectively.

We also studied the system by varying the rate of binding of miRNA to mRNA (c_3_), which was increased keeping the rate of production of the miRNA (c_1_) and rate of degradation of RISC (c_4_) fixed at appropriate level. One can anticipate that this rate plays a less important role in the overall dynamics and thus there is only a marginal change in the frequency of oscillation or in amplitude (somewhat more pronounced in Case 2) as can be seen in Fig. 6a, b. Similarly, when the rate of degradation of RISC (c_4_) was increased keeping c_1 _and c_3 _fixed, a marginal decrease in frequency and increase in amplitude was observed in Case 2, but in Case 1 there was no significant change (Figs. 7a, b). In all these studies, the miRNA decay rate (c_2_) was kept constant.

A similar exploration of the dynamics under variation of the parameters was undertaken for the corresponding deterministic model (see Additional Files 2, 3, 4 and 5). The results of both sets of studies are in good agreement qualitatively, and serve to confirm the robustness of these results, namely that under the effect of miRNA, a regulated protein can show both a significant reduction in expression level, as well as (in the present model) a decrease in the frequency of oscillation.

## Model B in Drosophila [20]

In this somewhat more elaborate model of the circadian rhythm, the periodicity of *Per *oscillations depends on the half-life of the mRNA and on its degradation rate. The involvement of miRNAs can effectively increase the degradation rate and thereby shorten the mRNA half-life.

### Case1: Degradation of mRNA in the RISC

**Figure 8:**
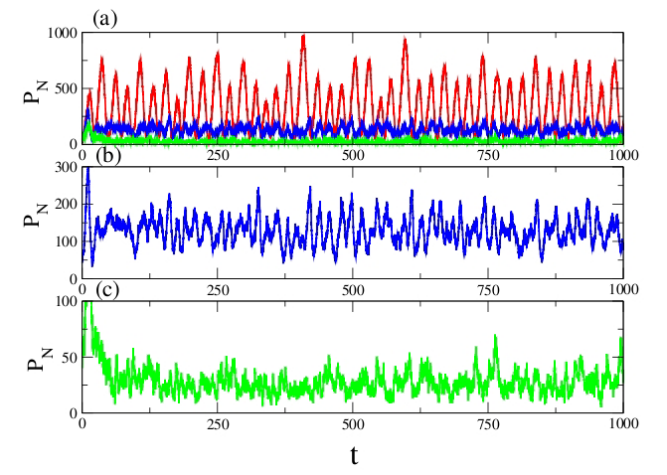
**Model B, Case 1: Temporal behaviour of the PER protein with miRNA regulation**. The degradation rate of the miRNA is kept constant at c_2 _= 0.029, and the volume is fixed at 100. In (a) the red curve shows the variation of PER in the absence of regulation, while the blue curve corresponds to regulation with c_1 _= 0.3 h^-1^, c_3 _= 0.08 h ^-1 ^and c_4 _= 0.01 h^-1^. The green curve has parameters c_1 _= 0.5 h^-1^, c_3 _= 0.1 h^-1 ^and c_4 _= 0.05 h^-1^. (b) and (c) are expanded views of (a).

The results of the simulation using different constants are shown in Fig. 8. While the introduction of miRNA drastically changes the amplitude of oscillations in this model (as in the simpler Model A), the effect on the frequency differs, increasing it somewhat.

### Case2: RISC releases miRNA and mRNA

**Figure 9:**
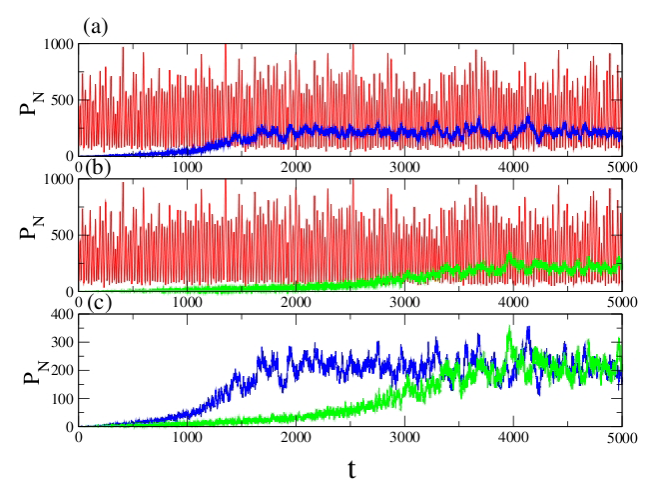
**Model B, Case 2: Temporal behaviour of the PER protein with miRNA regulation**. Degradation rate of the miRNA is fixed in all the cases and is taken to be c_2 _= 0.029. (a) Regulation with c_1 _= 10 h^-1^, c_3 _= 1 h^-1 ^and c_4 _= 0.5 h^-1^. (b) regulation with c_1 _= 15 h^-1^, c_3 _= 3 h^-1 ^and c_4 _= 1 h^-1^. (c) comparison of PER protein behaviour in (a) and (b). In all the cases, the volume of the system is taken to be 100.

On the other hand, when the mRNA and miRNAs were allowed to be released from the RISC without degradation, the frequency does not change as dramatically (Fig. 9), in comparison to the situation where degradation of miRNA and mRNA (Case1) takes place in RISC. As in model A, in this case the frequency appears to stabilize.

### The effect of parameter variation

**Figure 10:**
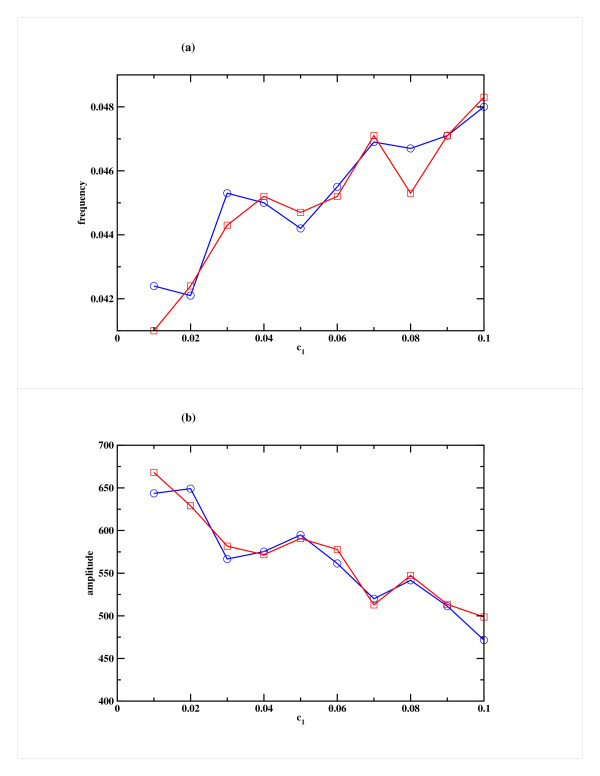
**Effect of variation of the miRNA production rate (c_1_) on the PER protein for Model B**. Variation of (a) the frequency and (b) the amplitude of oscillation in the PER protein as a function of c_1_, the rate of miRNA production. Cases 1 and 2 are shown in blue and red respectively. The other parameters, namely the rate of binding of miRNA to mRNA (c_3_) and RISC degradation rate (c_4_) are kept fixed at 0.02 h^-1 ^and 0.001 h^-1 ^respectively.

**Figure 11:**
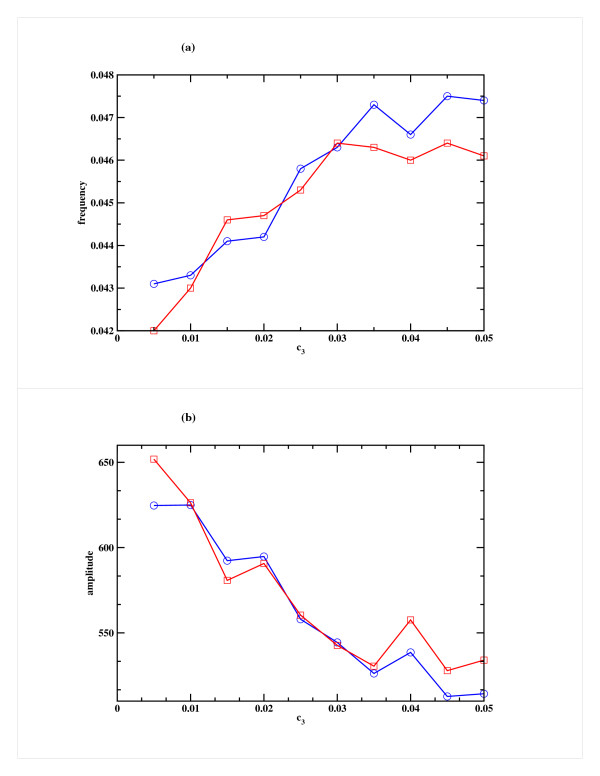
**Effect of variation of the rate of binding of miRNA to mRNA (c_3_) on the PER protein for Model B**. Variation of (a) the frequency and (b) the amplitude of oscillation in the PER protein as a function of c_3_, the rate of binding of the miRNA to mRNA. Cases 1 and 2 are shown in blue and red respectively. The other parameters, namely the miRNA production rate (c_1_) and RISC degradation rate (c_4_) are kept fixed at 0.05 h^-1 ^and 0.001 h^-1 ^respectively.

As for Model A, we have studied the change in the dynamics of the oscillator system when the different rates within the miRNA module are varied. The dynamics is most sensitive to variation of the miRNA production rate, c_1 _and to a smaller extent, the binding rate of the miRNA to the mRNA, namely c_3_. When these rates are increased, the main effect is to decrease the amplitude of oscillation, but the frequency of oscillation increases (see Figs. 10, 11). The corresponding deterministic model (see Additional Files 6, 7) also shows similar dynamics, supporting the observation that the main effect of miRNA regulation is to decrease the expression of the corresponding protein and to increase the frequency of its production in this model.

## Discussion

The regulation of cellular processes by miRNAs is in the initial stages of quantitative exploration, and it is not clear how it depends on the level of specific gene expression. The essential features of miRNA regulation are encapsulated in a simple four step stochastic processes, and we find that the essential features appear to be robust to parameter variation, and under conditions of both intrinsic and extrinsic noise. Simulations were also performed with varying cellular volume, in order to mimic cell doubling in 10 hours. The dynamics was not significantly different from that observed when the volume was kept fixed (data not shown here). Further, the essential qualitative features of the results are model independent, and thus miRNA regulation may be one of the primary means of controlling the period of oscillatory chemical and biochemical reactions within the cell. This may well be one of the principal strategies that enable the optimization of time-keeping within cellular and sub cellular processes.

It should be noted that other mechanisms can also be effective, as for example those outlined in recent studies by Xie et. al. [24] or Khanin and Vinciotti [25] who consider a deterministic model. We have also considered the deterministic limit of the above stochastic processes and find that there is a good correspondence of the present results and those that are obtained in the deterministic case (see Additional Files 1, 2, 3, 4, 5, 6 and 7).

## Conclusion

Gene expression in eukaryotes is controlled at different levels, and post-transcriptional processes play a significant role in many of these regulatory systems.

These include processing, transport, stability, sequestration and translation. In general many proteins are involved in such mechanisms.

In this paper we have explored the dynamics of regulation by miRNAs. While mRNA processing and degradation are also mediated through a number of RNAses present in cells, proteins in general are more stable than RNAs and it can be assumed that the concentrations of proteins remain at a constant level during the life of mRNAs. In this respect miRNA mediated regulation of gene expression is different as both molecules are labile. The overall dynamics of the system would be considerably different from that of protein-based mechanisms, for example translation is prevented by specific RNA-binding proteins that are known to bind secondary structure elements in UTRs [26]

Recent experimental studies have identified essentially two classes of mechanisms by which miRNAs are thought to regulate gene expression. Both of these involve post-transcriptional events that are translational inhibition and mRNA degradation, leading to inhibition in the amount of proteins produced. There are a large number of studies describing the effect of miRNA-based inhibition of gene expression on different biological processes. However, it is not clear how inhibition of one or a few genes has profound effect in biological decision-making. In this study, the mechanistic role of miRNAs has been explored using quantitative models and stochastic simulation. miRNAs have been incorporated in two different circadian oscillator models that were previously studied. The results of our analysis showed that the introduction of miRNAs not only changed the amplitude but also the frequency. This concurs with the recent discovery where two miRNAs were found to maintain mammalian circadian rhythms. The approach described here can be useful to analyse the influence of miRNAs on large genetic regulatory networks controlling fundamental biological processes.

## Methods

### Simulations

Time evolution of the oscillator models with the incorporation of miRNA is studied in the stochastic formalism via Monte Carlo simulations of the corresponding master equations through the direct Gillespie algorithm [27]. This was implemented in code developed by us. Details of the individual processes in each of the circadian oscillator models have been discussed extensively in earlier work [18,20] and the various parameters were chosen to correspond to the experimental systems discussed therein. We used the estimate given in recent work for the miRNA degradation rate [25]. Starting from a given initial configuration, the system dynamics is followed for a long period of time in order to remove transient behaviour. The stable oscillatory portion of this signal is identified, and Fourier transformation is then employed to extract the frequency of oscillations. The average amplitude of the oscillations is also calculated. Results are further ensemble averaged for a set of initial configurations.

## Authors' contributions

AN developed the model, ran the simulations and drafted the manuscript. CV advised on the model construction and contributed to the sections of the manuscript. AB and RR designed, supervised and coordinated the study. All authors read and approved of the final manuscript.

## Supplementary Material

Additional file 1 **"Study of the deterministic model"**. To complement our study of the stochastic model, an extensive parameter space exploration using the deterministic models was carried out. This file contains the deterministic formalism and the corresponding results. Click here for file

Additional file 2 **"Model A, Case1 and Case2: c_1 _– c_3 _: effect on Frequency"**. The c_1 _– c_3 _plot showing the effect on frequency in both the cases of Model A. Click here for file

Additional file 3 **"Model A, Case1 and Case2: c_1 _– c_3_: effect on Amplitude"**. The c_1 _– c_3 _plot showing the effect on amplitude in both the cases of Model A. Click here for file

Additional file 4 **"Model A, Case1 and Case2: c_1 _– c_4 _: effect on Frequency"**. The c_1 _– c_4 _plot showing the effect on frequency in both the cases of Model A. Click here for file

Additional file 5 **"Model A, Case1 and Case2: c_1 _– c_4 _: effect on Amplitude"**. The c_1 _– c_4 _plot showing the effect on amplitude in both the cases of Model A. Click here for file

Additional file 6 **"Model B, Case1 and Case2: c_1 _– c_3 _: effect on Frequency"**. The c_1 _– c_3 _plot showing the effect on frequency in both the cases of Model B. Click here for file

Additional file 7 **"Model B, Case1 and Case2: c_1 _– c_3 _: effect on Amplitude"**. The c_1 _– c_3 _plot showing the effect on amplitude in both the cases of Model B. Click here for file
