# Supplementary material for: miRNA-regulated dynamics in circadian oscillator models
Source: BMC Syst Biol. 2009 May 5;3:45. doi: 10.1186/1752-0509-3-45 (PMC2685780; doi:10.1186/1752-0509-3-45)
Supplement: Additional file 1 — "Study of the deterministic model". To complement our study of the stochastic model, an extensive parameter space exploration using the deterministic models was carried out. This file contains the deterministic formalism and the corresponding results. [file 1752-0509-3-45-S1.pdf]

Additional File 1

Study of the Deterministic model

In the limit of large volumes, the stochastic description of the reactions studied in this paper reduces to a set of coupled differential equations that correspond to the mass-action laws for the two models [18, 20]. To complement our study of the stochastic model, we have studied the corresponding deterministic systems extensively in the parameter space in order to verify that the results are robust. We present our results below; the main interest has been in seeing the variation of the frequency and amplitude as different parameters when miRNA production or RISC degradation were varied.

Ensembles of trajectories were integrated in each case and the average frequency of oscillation, F and average amplitude, A were extracted from the time-series by standard techniques. Contour plots were then constructed to facilitate easy qualitative analysis of the results.

We discuss the two miRNA regulated deterministic models below.

I. MODEL A

The deterministic relations for the first model are given by the following set of equations [18]:

$$\dot{D}_A = \theta_A D'_A - \gamma_A D_A A \tag{1}$$

$$\dot{D}_R = \theta_R D'_R - \gamma_R D_R A \tag{2}$$

$$\dot{D'}_A = \gamma_A D_A A - \theta_A D'_A \tag{3}$$

$$\dot{D'}_R = \gamma_R D_R A - \theta_R D'_R \tag{4}$$

$$\dot{M}_A = \alpha'_A D'_A + \alpha_A D_A - \delta_{MA} M_A \tag{5}$$

$$\dot{A} = \beta_A M_A + \theta_A D'_A + \theta_R D'_R - A(\gamma_A D_A + \gamma_R D_R - \gamma_C R + \delta_A) \tag{6}$$

$$\dot{M}_R = \alpha'_R D'_R + \alpha_R D_R - \delta_{MR} M_R \tag{7}$$

$$\dot{R} = \beta_R M_R - \gamma_C AR + \delta_A C - \delta_R R \tag{8}$$

$$\dot{C} = \gamma_C AR - \delta_A C. \tag{9}$$

where standard notation is used for the time derivative, and the parameters are as given in the text. When miRNA regulation (Eqs. (1-4) in the paper) is incorporated in the above model, the dynamical equation for M_R now has an additional term, and the dynamical equations for the miRNA (m) and RISC complex C_{RISC} are to be included.

$$\dot{M}_R = \alpha'_R D'_R + \alpha_R D_R - \delta_{MR} M_R - c_3 m M_R \tag{10}$$

$$\dot{m} = c_1 - c_2 m - c_3 m M_R \tag{11}$$

$$\dot{C}_{RISC} = c_3 m M_R - c_4 C_{RISC} \tag{12}$$

In Case 2, when the RISC dissociates to release miRNA and mRNA, the above equations are modified to

$$\dot{M}_R = \alpha'_R D'_R + \alpha_R D_R - \delta_{MR} M_R - c_3 m M_R + c_4 C_{RISC} \tag{13}$$

$$\dot{m} = c_1 - c_2 m - c_3 m M_R + c_4 C_{RISC} \tag{14}$$

$$\dot{C}_{RISC} = c_3 m M_R - c_4 C_{RISC} \tag{15}$$

II. Results for MODEL A.

The parameters c_1, c_3 and c_4 were varied in pairs and contour plots of frequency and amplitude variation were obtained for both Cases 1 and 2. Additional Files 2 and 3 show the results for variation of c_1 and c_3, while Additional Files 4 and 5 correspond to variation of c_1 and c_4. There was no significant variation in the dynamics when the parameters c_3 and c_4 were varied (keeping c_1 fixed at an intermediate value) and therefore we do not discuss this case.

As can be seen in Case 1, a major role is played by the rate c_1 which determines how miRNA is introduced into the system. The frequency shows a decrease as c_1 increases. This is similar to the results obtained using the stochastic model. c_3, the rate of binding of miRNA to mRNA, appears to play no role: the frequency and amplitude stay roughly constant as this rate is varied. The amplitude varies in a manner similar to the frequency (see Additional Files 2 and 3).

In Case 2, on the other hand, while the frequency decreases with increasing c_1, the effect is less pronounced than in Case 1: the frequency shows a decrease over a narrow range. When c_3 is varied, the frequency appears to increase, in concordance with the stochastic simulations. The amplitude, however, decreases with increase in parameters.

Shown in Additional File 4 is the variation in the frequency when c_1 and c_4 were varied. There is a strong dependence on c_1 and slight on c_4 as in the case of c_3. In

Case 2, however, the variation of c_4 has a more pronounced effect, showing an interesting variation of frequency, although the amplitude does not appear to vary significantly over this range. The amplitude decreases as a function of c_1 in Case 1, but in Case 2, there is actually a slight increase in the amplitude when c_4 is increased: this is due to the increase of mRNA when the RISC dissociates (Additional File 5).

III. MODEL B.

The deterministic model for the circadian oscillator developed by Goldbeter and coworkers [20] can be obtained from the chemical reactions. [20]. Incorporating miRNA as above, gives the following set of additional equations for Case 1.

$$\dot{M}_P = v_s \frac{K^n_I}{K^n_I + P^n_N} - v_m \frac{M_p}{K_m + M_p} - c_3\, mM_P \quad (16)$$

$$\dot{m} = c_1 - c_2 m - c_3\, mM_P \quad (17)$$

$$\dot{C}_{RISC} = c_3\, mM_P - c_4\, C_{RISC} \quad (18)$$

while for Case 2, they are modified to

$$\dot{M}_P = v_s \frac{K^n_I}{K^n_I + P^n_N} - v_m \frac{M_p}{K_m + M_p} - c_3\, mM_P + c_4 C_{RISC} \quad (19)$$

$$\dot{m} = c_1 - c_2 m - c_3\, mM_P + c_4 C_{RISC} \quad (20)$$

$$\dot{C}_{RISC} = c_3\, mM_P - c_4\, C_{RISC} \quad (21)$$

IV. Results for MODEL B.

As above for Model A, the parameters c_1, c_3 and c_4 were varied in pairs and contour plots of frequency and amplitude variation were obtained for both Cases 1 and 2.

Additional Files 6 and 7 show the results for variation of c_1 and c_3. We discuss the dynamical variation for the other combinations in brief.

Here too, the main parameter that controls the dynamics is c_1. However, in contrast to Model A, the frequency increases with c_1 in a manner analogous to the stochastic simulations. The amplitude, however, decreases. The variation with c_3 is less pronounced.

When c_1 and c_4 were changed, keeping c_3 fixed, the behaviour is very similar (although not as pronounced) as in the case above. When c_1 is fixed and c_3 and c_4 were varied, the effect is also marginal: the frequency increases with increasing rates, while the amplitude decreases.

V. Discussion

In summary, the deterministic simulations support the dynamical behaviour seen in the stochastic studies of the two circadian oscillator models with miRNA based regulation. Although the deterministic equations are somewhat simpler to analyse than the master equation corresponding to the stochastic model, these are of sufficiently high dimension so as to preclude a straightforward analysis. These, however, allow for a more extensive exploration of parameter space that we have carried out here, and complement the stochastic studies inasmuch as the results of the two are in consonance, showing that the major effect of the miRNA is to *reduce* the amplitude of oscillations of the controlled protein, namely to down-regulate the gene while affecting the frequency of oscillations, decreasing it in Model A, and increasing it in Model B. In either case, this would have the effect of altering cellular processes by changing time keeping system.
