# Supplementary figures and images for: miRNA-regulated dynamics in circadian oscillator models
Source: BMC Syst Biol. 2009 May 5;3:45. doi: 10.1186/1752-0509-3-45 (PMC2685780; doi:10.1186/1752-0509-3-45)

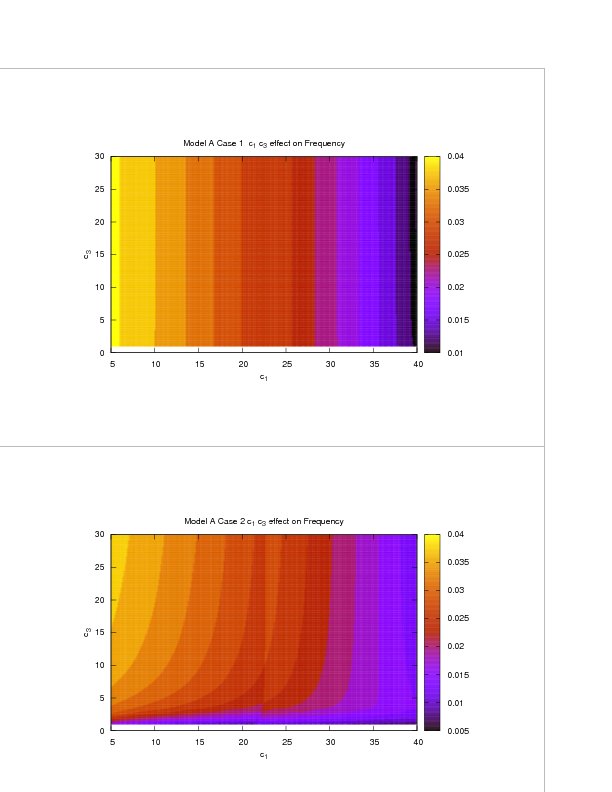

Supplement: Additional file 2 — "Model A, Case1 and Case2: c1 – c3 : effect on Frequency". The c1 – c3 plot showing the effect on frequency in both the cases of Model A. [file 1752-0509-3-45-S2.jpeg]

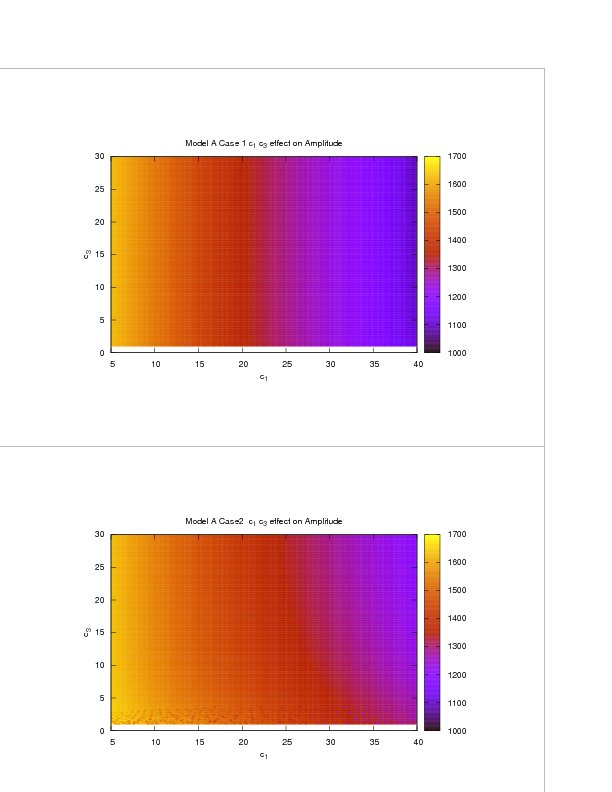

Supplement: Additional file 3 — "Model A, Case1 and Case2: c1 – c3: effect on Amplitude". The c1 – c3 plot showing the effect on amplitude in both the cases of Model A. [file 1752-0509-3-45-S3.jpeg]

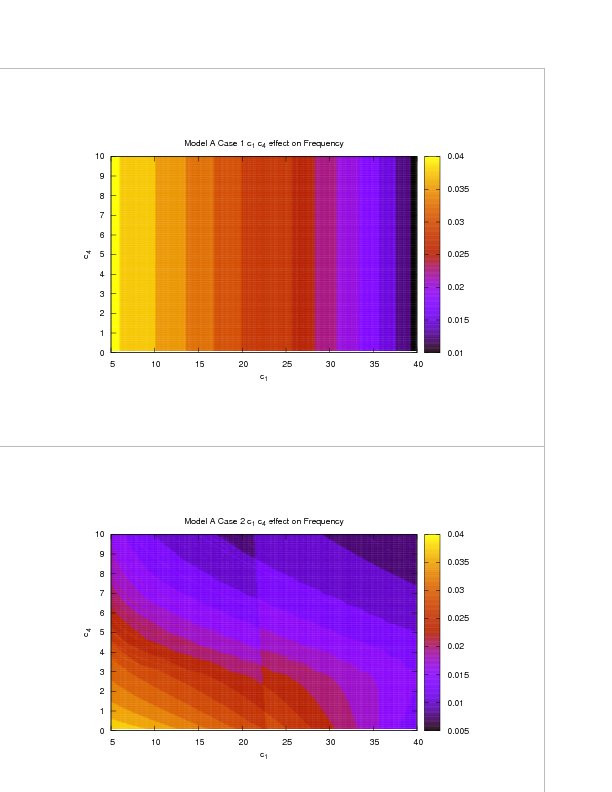

Supplement: Additional file 4 — "Model A, Case1 and Case2: c1 – c4 : effect on Frequency". The c1 – c4 plot showing the effect on frequency in both the cases of Model A. [file 1752-0509-3-45-S4.jpeg]

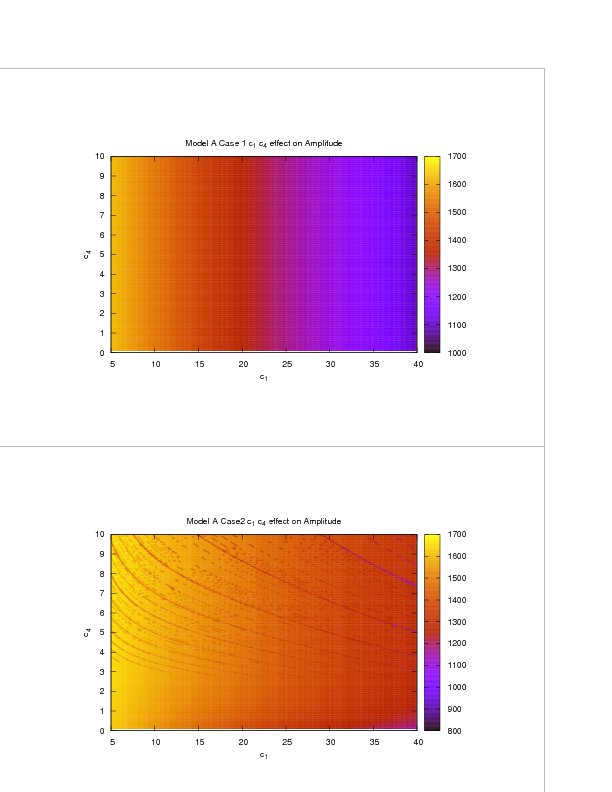

Supplement: Additional file 5 — "Model A, Case1 and Case2: c1 – c4 : effect on Amplitude". The c1 – c4 plot showing the effect on amplitude in both the cases of Model A. [file 1752-0509-3-45-S5.jpeg]

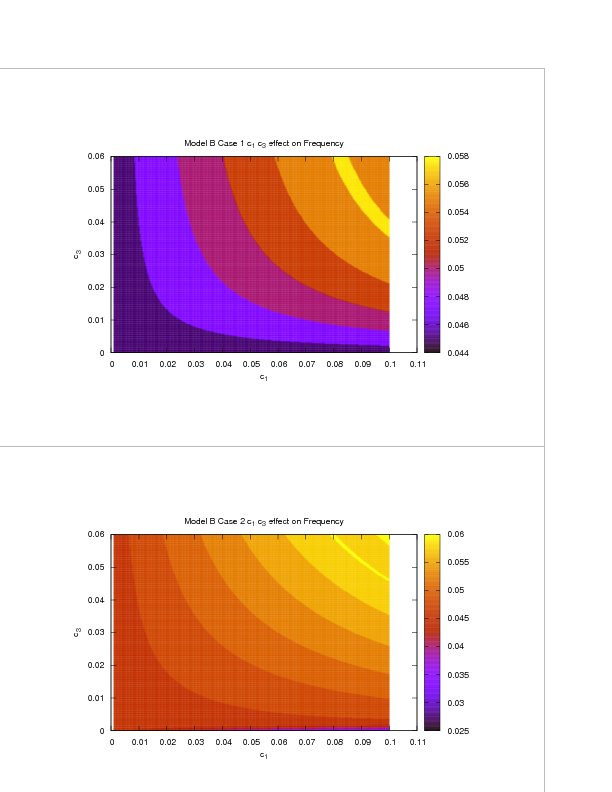

Supplement: Additional file 6 — "Model B, Case1 and Case2: c1 – c3 : effect on Frequency". The c1 – c3 plot showing the effect on frequency in both the cases of Model B. [file 1752-0509-3-45-S6.jpeg]

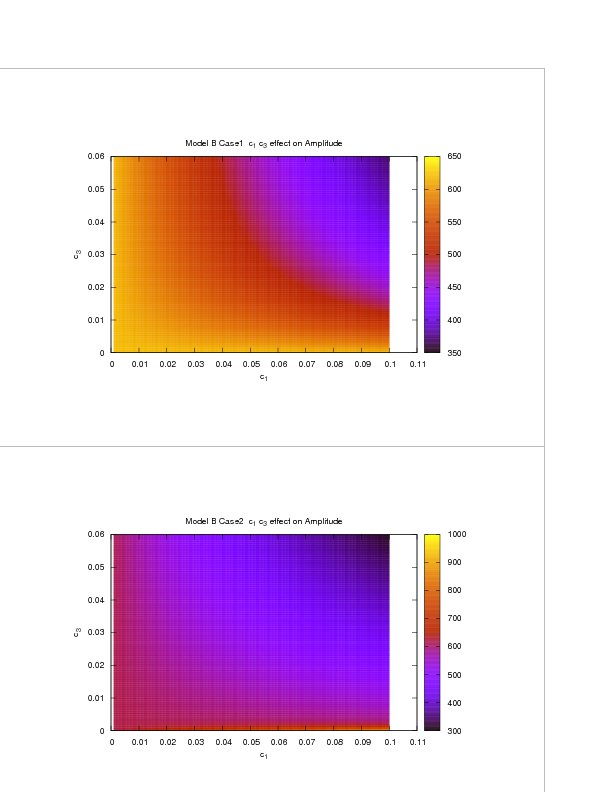

Supplement: Additional file 7 — "Model B, Case1 and Case2: c1 – c3 : effect on Amplitude". The c1 – c3 plot showing the effect on amplitude in both the cases of Model B. [file 1752-0509-3-45-S7.jpeg]
